# Altered Default Mode Network Functional Connectivity in Parkinson’s Disease: A Resting-State Functional Magnetic Resonance Imaging Study

**DOI:** 10.3389/fnins.2022.905121

**Published:** 2022-06-03

**Authors:** Lu Chen, Ting Huang, Di Ma, Yu-Chen Chen

**Affiliations:** ^1^Department of Radiology, Nanjing Integrated Traditional Chinese and Western Medicine Hospital Affiliated With Nanjing University of Chinese Medicine, Nanjing, China; ^2^Department of Neurology, Nanjing First Hospital, Nanjing Medical University, Nanjing, China; ^3^College of Information Science and Technology, Nanjing Forestry University, Nanjing, China; ^4^Department of Radiology, Nanjing First Hospital, Nanjing Medical University, Nanjing, China

**Keywords:** Parkinson’s disease, cognitive decline, functional connectivity, default mode network, functional magnetic resonance imaging

## Abstract

**Purpose:**

Whether the intrinsic functional connectivity pattern of the default mode network (DMN) is involved in the progression of cognitive decline in Parkinson’s disease (PD) remains unclear. This study aimed to investigate the intrinsic functional connectivity (FC) pattern of the DMN anchored on the posterior cingulate cortex (PCC) in patients with PD by resting-state functional magnetic resonance imaging (fMRI).

**Methods:**

Fifty patients with PD and 50 healthy controls (HCs) were included for resting-state fMRI scanning. A seed-based FC method was used to reveal FC patterns in the DMN with region of interest (ROI) in the PCC. Relationships between FC patterns and disease severity (UPDRS-III) were detected.

**Results:**

Compared with the HCs, the patients with PD showed increased FC between the PCC and the right precuneus, left cuneus, and right angular gyrus. In the PD group, the increased FC values in the right precuneus were significantly and positively correlated with motor severity as assessed with UPDRS-III scores (rho = 0.337, *p* = 0.02).

**Conclusion:**

Our result highlights that the patients with PD showed increased FC between the PCC and the right precuneus, left cuneus, and right angular gyrus in the DMN. The altered connectivity pattern in the DMN may play a crucial role in the neurophysiological mechanism of cognitive decline in patients with PD. These findings might provide new insights into neural mechanisms of cognitive decline in PD.

## Introduction

Parkinson’s disease (PD) is a progressive neurodegenerative disorder that is characterized by movement disturbances such as bradykinesia, resting tremor, rigidity, and postural instability (Nemade et al., 2021; Tolosa et al., 2021). According to a survey in Europe, the prevalence rate of PD is 65.6 per 100,000–12,500 per 100,000 (Bloem et al., 2021; Le Heron and Macaskill, 2021). PD is not only accompanied by motor disorder, but it also leads to cognitive decline. Ding et al. demonstrated that three cognitive impairments, namely, executive function deficits, memory deficits, and visuospatial deficits, are most commonly observed in patients with PD (Ding et al., 2015). Stav et al. (2015) reported that lower amyloid-β42 in cerebrospinal fluid biomarkers was a potential biomarker for cognitive decline functions in early PD, mainly connected to medial temporal lobe-based cognitive functions. Besides, patients with PD are at high risk for dementia (Hanagasi et al., 2017), and the etiology of the neurophysiological mechanism of this increased risk remains unclear.

Recent advances in functional magnetic resonance imaging (fMRI) approaches have provided a non-invasive method for characterization of central nervous system changes in some specific brain regions in PD (Mitchell et al., 2021), including the basal ganglia (Szewczyk-Krolikowski et al., 2014; Rolinski et al., 2015; Filyushkina et al., 2019), hippocampus (Zeng et al., 2017; Guo et al., 2021), cingulate gyrus (Zhang et al., 2017; Wang et al., 2021), and so on. Zhang et al. (2017) found that patients with PD and fatigue had amplitude of low-frequency fluctuation (ALFF) changes in the right middle frontal gyrus within the attention network and in the left insula as well as right middle cingulate gyrus within the salience network. Meanwhile, Hou et al. (2014) demonstrated that patients with PD had decreased ALFF in the striatum and increased ALFF in the midbrain in slow-4 (0.027–0.073 Hz) relative to HCs. Moreover, Zeng et al. (2017) demonstrated that patients with PD showed decrease in regional homogeneity (ReHo) in the sensorimotor cortex, default mode network, and left cerebellum but increased ReHo in the supplementary motor area, bilateral temporal gyrus, and hippocampus with disease progression. Furthermore, PD also leads to brain network dysfunction. Various neuroimaging studies demonstrated that basal ganglia network dysfunction played an important role in impaired motor function in patients with PD (Filyushkina et al., 2019). Rolinski et al. demonstrated that decreased connectivity in the basal ganglia was detected in patients with PD (Rolinski et al., 2015). Szewczyk-Krolikowski et al. (2014) reported that patients with PD were associated with decreased functional connectivity in the basal ganglia network relative to HCs. However, these findings mainly focused on the abnormal local neural activity and basal ganglia network in patients with PD. Few studies on neurocognitive network alterations in patients with PD, especially the default mode network (DMN), have been reported.

The human brain is a complex dynamic system capable of generating low-frequency oscillations (LFOs) at rest (Wise et al., 2004). Low-frequency fluctuations (< 0.01 Hz) in blood oxygenation level-dependent signals during rest reflect spontaneous neural activity, which can be conceptualized as a network of anatomically linked regions. The DMN is regarded as an endogenous neural network that shows consistently higher blood oxygenation level-dependent activity during rest. The DMN shows consistently higher BOLD activity during rest in several brain regions such as the middle temporal gyrus (MTG), medial prefrontal cortex (mPFC), posterior cingulate cortex (PCC), anterior cingulate cortex, and inferior parietal lobe (Greicius et al., 2003). The DMN plays an important role in various higher cognitive functions such as memory, prospection, and self-processing (Spreng and Grady, 2010). The PCC is a key region of the DMN with strong connectivity in primates with entorhinal cortex, parahippocampal gyrus, and, thus, hippocampal memory system, which is involved in autobiographical memory and imagining the future; and spatial navigation and scene processing (Leech and Sharp, 2014). Several prior studies have reported disrupted functional connectivity of the DMN in patients with PD by resting-state fMRI (Guo et al., 2021; Shang et al., 2021b; Wang et al., 2021) or fluorodeoxyglucose positron emission tomography (FDG-PET) (Ruppert et al., 2021; Schindlbeck et al., 2021; Shang et al., 2021a). However, how DMN dysfunction draws a relationship to cognitive symptoms in patients with PD still remains unknown.

To address this issue, we performed a voxel-level resting-state functional-connectivity neuroimaging analysis of the PCC with all other voxels in the brain between groups. This study aimed to determine whether patients with PD with impaired cognition exhibited an abnormal functional connectivity (FC) pattern of the DMN using a seed-based approach. Moreover, we investigated relationships between abnormal functional connectivity in the DMN and clinical variables in the PD group. Our findings might provide new insights into neural mechanisms of cognitive decline in PD.

## Materials and Methods

### Participants

Fifty patients with PD were enrolled from the Department of Neurology Affiliated to Nanjing First Hospital. The diagnostic criteria of PD are according to the clinical criteria of Movement Disorder Society (Postuma et al., 2015). For the PD group, disease severity and stage were evaluated using the Unified Parkinson’s Disease Rating Scale part III (UPDRS-III) and Hoehn and Yahr (H-Y) scale, respectively. Interviews and clinical assessments were conducted structurally by two experienced neurologists.

The exclusion criteria for all the participants were as follows: (1) family history of PD, dementia, or hypertension; (2) complaint of cognitive impairment with MMSE score < 27 or MoCA score < 26; (3) additional neuropsychological disorders (e.g., Alzheimer’s disease, schizophrenia, depression, and epilepsy) or cerebrovascular accidents (e.g., stroke and intracranial hemorrhage); (4) any complications or lesions involving the central nervous system (e.g., diabetes mellitus, hyperthyroidism, tumor, and brain trauma); (5) history of alcoholism or substance abuse; any condition contraindicating MRI scanning (e.g., metal foreign body or implant, claustrophobia, and hyperpyrexia) or inducing severe head movement (e.g., hearing or visual loss); (6) years of education < 9. Fifty age- and gender-matched healthy controls (HCs) were also recruited for this study. Studies involving human participants are reviewed and approved according to the Declaration of Helsinki and by the institutional review board of Nanjing Medical University. The patients/participants provided their written informed consent to participate in this study.

All the patients with PD underwent a cognitive assessment, including global cognitive tests and an extensive neuropsychological test battery, to assess the neurocognitive state. The global cognitive assessments contained the Montreal Cognitive Assessment (MoCA) (Nasreddine et al., 2005).

### Magnetic Resonance Imaging Acquisition

The subjects were scanned under resting conditions using a 3.0 Tesla MRI scanner (MAGNETOM Prisma; Siemens Healthcare, Erlangen, Germany) equipped with a 64-channel receiver array head coil. During scanning, the subjects were supposed to lie quietly with their eyes closed and avoid head movement but not to fall asleep or think about anything special. To reduce head motion and scanner noise, foam pads and earplugs were used. Resting-state functional images were obtained axially using a gradient echo-planar imaging (EPI) sequence as follows: repetition time (TR) = 2,000 ms, echo time (TE) = 30 ms, slices = 33, thickness = 4 mm, gap = 0 mm, field of view (FOV) = 192 mm × 192 mm, acquisition matrix = 64 × 64, and flip angle (FA) = 90°. Recently, simultaneous multi-slice (SMS) imaging techniques have been used to highly accelerate the time of acquisition (Feinberg and Setsompop, 2013; Chen et al., 2015). Thus, SMS-accelerated EPI reconstruction was applied in this study. Structural images were obtained using a magnetization-prepared rapid gradient echo (MP2RAGE) sequence and following scan parameters: TR/TE = 5,000/2.98 ms, slices = 176, thickness = 1 mm, gap = 0 mm, FA = 90°, acquisition matrix = 256 × 256, and FOV = 256 mm × 256 mm.

### Data Processing

The functional images were preprocessed using Statistical Parametric Mapping SPM12^1^ and the toolbox for Data Processing Assistant and Analysis for Brain Imaging (DPABI)^2^ implemented in MATLAB 2013b (MathWorks, Natick, MA, United States) and in brief the following steps: (1) remove the first ten volumes function images, slice timing effects and head motion corrected; (2) Individual T1-weighted MP2RAGE structural images were registered to the mean fMRI data (Yan et al., 2016). Normalized data in Montreal Neurological Institute (MNI) 152 space were reliced at a resolution of 3 × 3 × 3mm^3^ and spatially smoothed with an 8-mm full width at half-maximum Gaussian kernel; (3) The linear regression to reduce the contribution of non-neuronal fluctuations; (4) Band-pass was filtered (0.01–0.08 Hz).

### Definition of the Seed Region of Interest and Functional Connectivity Analysis

According to previous studies (Goto et al., 2013), the coordinate is the posterior cingulate cortex (PCC) (*x* = 0, *y* = −53, *z* = 26) and is 6 mm in diameter. For seed-based FC analysis, a correlation analysis of time course was performed between the spherical seed region (PCC) and each voxel of the whole brain for each subject using the DPABI software.

### Statistical Analysis

The normality distribution of demographic characteristics and clinical assessments was checked using the Kolmogorov–Smirnov method. The intergroup difference of age was analyzed by using independent-sample *t*-test. The comparison of sex between PD and HCs was conducted by χ^2^-test. Mann–Whitney U test was applied for comparisons between groups in years of education and scores of clinical scales. SPSS software (version 20.0, SPSS Inc., Chicago, IL, United States) was utilized for the above statistical analyses. A *p*-value < 0.05 was defined as statistically significant.

One-sample *t*-test was conducted to assess patterns of DMN maps using the DPABI software. Two-sample *t*-test was conducted to assess different zFC maps between groups using the Gaussian random field (GFR) method, which was used to correct for multiple comparisons using the DPABI software (Two-tailed, voxel-level: *p* < 0.01, GRF correction, cluster-level: *p* < 0.05). Finally, Pearson correlation coefficients between altered zFC values and clinical variables were analyzed using SPSS.

## Results

### Demographics Measurements

The detailed demographic characteristics and clinical assessments are shown in Table 1. The patients with PD were included at the early stage (H-Y stage, mean 1.41 ± 0.45) with a relatively short disease duration (mean 6.32 ± 4.63 years). No significant differences in age, gender, and years of education were observed between the patients with PD and the HCs (*p* > 0.05). The PD group had lower MoCA scores than the HCs (*p* < 0.001).

**TABLE 1:** Demographic and clinical characteristics of patients with PD and HCs.

	HCs (*n* = 50)	PD (*n* = 50)	*T/Z*	*P*
Age	61.74 ± 6.17	62.88 ± 9.06	0.735	0.464
Sex (M/F)	30/20	32/18	0.170	0.680
Education (years)	10.44 ± 3.68	10.26 ± 3.30	−0.257	0.797
Disease duration (years)	–	6.32 ± 4.63	–	–
UPDRS-III	–	27.76 ± 11.79	–	–
H-Y stage MoCA scores	– 28.20 ± 1.32	1.41 ± 0.45 24.36 ± 2.86	– −8.608	– <0.001

*Data are represented as mean ± standard deviation. HC, healthy control; PD, Parkinson’s disease; M, male; F, female; UPDRS, Unified Parkinson’s Disease Rating Scale; H-Y, Hoehn-Yahr.*

### Functional Connectivity Differences of the Default Mode Network

#### The Spatial Pattern of Default Mode Network Between Parkinson’s Disease Group and Healthy Controls Group

**FIGURE 1:**
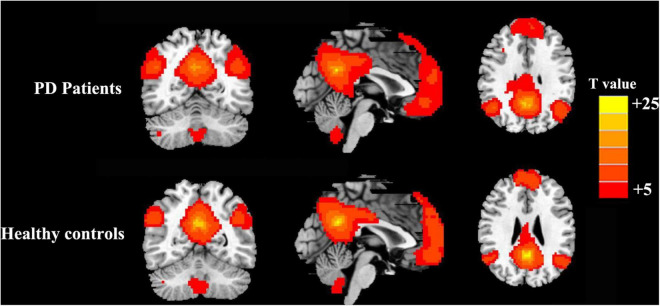
Results of the two components representing the default mode network (DMN) by one-sample *t*-test in patients with Parkinson’s disease (PD) and healthy controls.

**FIGURE 2:**
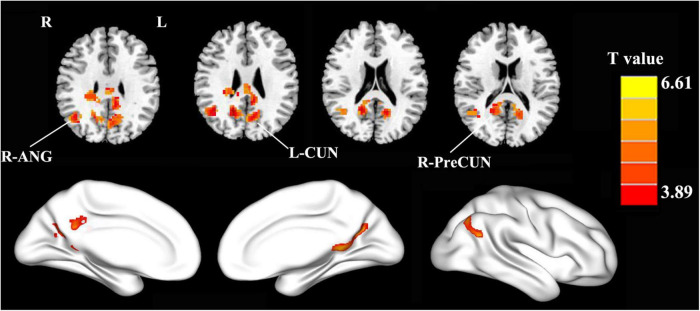
Compared with the healthy controls, the patients with PD exhibited enhanced FC between the PCC and the right precuneus (R-PreCUN), left cuneus (L-CUN), and right angular gyrus (R-ANG) (two-tailed, voxel-level: *p* < 0.01, GRF correction, cluster-level: *p* < 0.05).

The PCC mainly showed positive FC in DMN regions, including the mPFC, inferior parietal lobule (IPL), and precuneus (Figure 1). Compared with the HCs, the patients with PD showed increased FC between the PCC and the right precuneus (PreCUN), left cuneus (CUN), and right angular gyrus (ANG) (Figure 2 and Table 2).

**TABLE 2:** Brain region with significant difference in FC values between patients with PD and HCs.

Brain regions	Peak MNI coordinates			Peak *t*-value		Cluster size (number of voxels)
	X	Y	Z		BA	
**PD > HC**						
Right precuneus Left cuneus right angular gyrus	16 −16 45	−50 −54 −60	28 24 39	6.51 5.26 6.03	23 19 39	236 257 153

*The statistical threshold was set at the voxel level with p < 0.01 for multiple comparisons using Gaussian random-field theory (two tailed, voxel-level p < 0.01, GRF correction, cluster-level p < 0.05). FC, functional connectivity; BA, Brodmann area; MNI, Montreal Neurological Institute; PD, Parkinson’s disease; HCs, healthy controls.*

### Correlation Analysis

**FIGURE 3:**
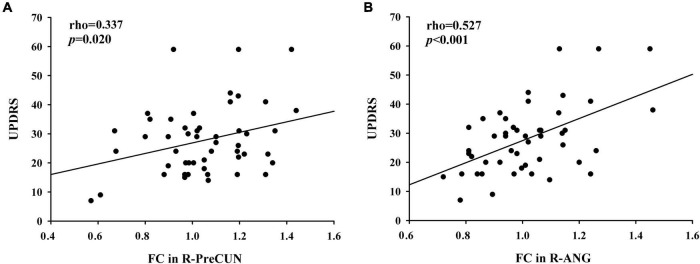
**(A)** Positive correlations between enhanced functional connectivity (FC) between the PCC and the right PreCUN and UPDRS-III scores in patients with PD (rho = 0.337, *p* = 0.02). **(B)** Moreover, the enhanced FC values in the right ANG were positively associated with the Unified Parkinson’s Disease Rating Scale part III (UPDRS-III) scores (rho = 0.527, *p* < 0.001).

After the correlation analysis, in the PD group, the increased FC values in the right PreCUN were significantly positively correlated with motor severity as assessed with UPDRS-III scores (rho = 0.337, *p* = 0.02) (Figure 3A). Moreover, the enhanced FC values in the right ANG were also positively associated with the UPDRS-III scores (rho = 0.527, *p* < 0.001) (Figure 3B).

## Discussion

This study examining the DMN in patients with PD has generated several important results. The main findings were that the patients with PD showed increased FC between the PCC and the right PreCUN, left CUN, and right ANG relative to the HC group. Moreover, these abnormalities were significantly correlated with the disease severity of PD. In our study, we found that PD patients had higher FC between the PCC and the right PreCUN relative HC. The PreCUN is the core component of the DMN (Yu et al., 2017). The PreCUN plays an important role in self-centered mental imagery and episodic memory retrieval (Utevsky et al., 2014). Previous neuroimaging studies demonstrated that PreCUN dysfunction was observed in patients with PD, which is involved in memory deficits, attention and working memory deficits, and time perception deficits in patients with PD. The increased FC between the PCC and the PreCUN was also observed in other neurodegenerative diseases including Alzheimer’s disease (Churchill et al., 2021). In line with these findings, we speculated that enhanced FC between the PCC and the right PreCUN might indicate cognition dysfunction in patients with PD.

We also found that the patients with PD showed increased FC between the PCC and the right ANG relative to the HCs. Previous neuroimaging studies demonstrated that ANG plays an important role in semantic information processing (Boylan et al., 2015). Meanwhile, the ANG is also involved in mnemonic functions (Matchin et al., 2019). Moreover, the ANG is the core hub of the DMN (Ramanan et al., 2018). It was shown that lack of desynchronization of brain oscillatory activity in the PCC and right ANG disrupts the efficient processing in the fronto-parietal working memory network, leading to decline in visual working memory performance (Vatansever et al., 2017). Thus, we speculated that the increased FC between the PCC and the right ANG might indicate the working memory performance in patients with PD. Furthermore, in the PD group, the increased FC values in the right Pre CUN and right ANG were significantly positively correlated with the disease severity of PD. Li et al. observed that altered global synchronizations in DMN regions such as the IPL were correlated with UPDRS-III scores, which was similar with our current results (Li M. et al., 2019). The DMN is also thought to be associated with self-referential processing (Gusnard et al., 2001; Li W. et al., 2019). We hypothesized that higher global synchronizations of the DMN in PD may result in decreased ability to be self-referential, more likely to maintain the default mode state, and less control of interactions between brain regions. Therefore, increased FC in the DMN might be a potential biomarker for identifying neural mechanism dysfunction in patients with PD.

Several limitations must be acknowledged in our study. First, previous longitudinal data have indicated a dissociation of the DMN in PD, where reduced connectivity between the PCC and the mPFC is associated with future cognitive impairments (Zarifkar et al., 2021). However, our study is cross-sectional with a relatively small sample size. Thus, it is difficult to make direct causal inferences regarding the relationships between the aberrant FC and the disease severity of PD. Further longitudinal fMRI studies are required to establish the causal relationships and confirm the current findings. Second, our study lacked an assessment of various neurophysiological tests on patients with PD. Moreover, the choice of seed may bias connectivity findings toward specific, smaller, or overlapping sub-systems rather than larger distinct networks (Buckner et al., 2008). Some data-driven fMRI techniques, such as independent component analysis (ICA) or graph theory analysis, will be utilized in our future study. Finally, physiologic noise including respiratory, head motion, and cardiac fluctuations, might have compromised our results. These confounding factors should be taken into consideration in future studies.

## Conclusion

This study highlights that patients with PD showed aberrant FC in the DMN. The enhanced connectivity pattern in the DMN may play a pivotal role in the neurophysiological mechanism of cognitive decline in patients with PD.

## Data Availability Statement

The original contributions presented in the study are included in the article/supplementary material, further inquiries can be directed to the corresponding author/s.

## Ethics Statement

The studies involving human participants were reviewed and approved by the institutional review board of Nanjing Medical University. The patients/participants provided their written informed consent to participate in this study.

## Author Contributions

LC and TH drafted the manuscript for the work. DM helped to acquire the clinical and fMRI data. Y-CC took care of the financial support, review, and final approval of the manuscript to be published. All authors have read and approved the final manuscript.

## Conflict of Interest

The authors declare that the research was conducted in the absence of any commercial or financial relationships that could be construed as a potential conflict of interest. The reviewer ZX declared a past co-authorship with the author Y-CC to the handling editor.

## Publisher’s Note

All claims expressed in this article are solely those of the authors and do not necessarily represent those of their affiliated organizations, or those of the publisher, the editors and the reviewers. Any product that may be evaluated in this article, or claim that may be made by its manufacturer, is not guaranteed or endorsed by the publisher.

## Footnotes

1
http://www.fil.ion.ucl.ac.uk/spm/

2
http://rfmri.org/dpabi
